# A Study on the Effect of the Pre-Go-Live Training in Anxiety and Depression of Medical Staff Based on the Data of Wuhan Fangcang Shelter Hospital During COVID-19 in the Era of Big Data

**DOI:** 10.3389/fpubh.2022.909241

**Published:** 2022-05-31

**Authors:** Qiang Feng, Huizhi Zhou, Lu Wang, Chuanyuan Kang

**Affiliations:** ^1^Department of Psychosomatic Medicine, Shanghai East Hospital, Tongji University School of Medicine, Shanghai, China; ^2^Tongji University School of Medicine, Shanghai, China

**Keywords:** COVID-19, Fangcang shelter hospital, mental health care, health psychology, public health

## Abstract

Coronavirus disease 2019 (COVID-19) broke out in 2019. In the past 4 years, China has adopted many measures to control the epidemic, including building Fangcang shelter hospitals to isolate confirmed positive cases. Therefore, we aim to explore the mental health status of medical staff in the Wuhan Fangcang shelter hospital and discuss the relevant factors that affect the medical staff's mental status. The subjects of the research were staff from several Fangcang shelter hospitals in Wuhan during the epidemic of COVID-19. Patient Health Questionnaire−9 items Scale (PHQ-9) was used to assess the severity of the participants' depressive symptoms, and Generalized Anxiety Disorder−7 items Scale (GAD-7) was used to evaluate the severity of the participants' anxiety symptoms. The demographic information and health adjustment methods were collected in a self-made questionnaire, and regression analysis on related factors that affect mental health was performed. The three most frequently used methods of psychological adjustment for the staff in the Fangcang shelter hospital are common recreational activities, such as reading, streaming videos, listening to music, and playing games. (93.8%), communicating with colleagues in the Fangcang shelter hospital (92.5%), and communicating with family members and friends (78.3%). Binary logistic regression analysis showed that developing depression symptoms has relation to 2 factors, which are having not participated in medical emergency rescue missions (odds ratio = 2.610; 95% confidence interval 1.398–4.872, *P* = 0.003) and inadequate training before entering the shelter hospital (odds ratio = 2.804, 95% confidence interval 1.293–6.08, *P* = 0.009). Compared with adequate pre-job training, insufficient training increases the risk of anxiety symptoms (odds ratio = 2.692; 95% confidence interval 1.3–5.575, *P* = 0.008). Lack of experience and inadequate training in medical emergency rescue missions exposed the medical staff to a higher risk of developing symptoms of depression and anxiety. Psychological adjustment methods that are helpful to adjust their mental state are most commonly used.

## Introduction

With the development of technology, big data has been more frequently used in people's life. By the end of December 2019, the coronavirus pneumonia (COVID-19) (1) was spreading domestically in Wuhan city of China, which quickly became the epicenter of the COVID-19 pandemic. Under the circumstance of shortage of hospital beds, Wuhan Fangcang shelter hospitals were designed and provided for the first time in China to tackle the COVID-19 outbreak and offer medical care for patients with mild or moderate COVID-19 (2). Within a few months, Wuhan opened a whopping 16 Fangcang shelter hospitals equipped with over 13,000 hospitals beds (3).

Under such a crisis situation, healthcare providers on the frontline caring for patients positive for COVID-19 were prone to developing psychological distress and mental symptoms. Recent research shows that medical workers caring for patients with COVID-19, especially nurses and women, in fever clinics and wards in Wuhan and other regions in China reported experiencing more psychological stress (4), and that they experienced the challenges of daily clinical work in COVID-19 wards (5–7). As the epidemic of COVID-19 progressed, local healthcare workers in Wuhan became overworked under the situation of the accumulative number of suspected and confirmed clinical cases in the process of the outbreak. In response to the medical crisis, 42,000 healthcare workers (including 28,600 nurses) across pan-China came to Wuhan's aid, including neighboring cities within Hubei province (8). Although it was a joint effort in an unfamiliar environment full of challenges, medical team members from their respective hospitals are kept intact during the Fangcang shelter tenure (9, 10). In addition, professional support from psychiatrists who consistently conducted face-to-face group psychological interventions and online psychological services for medical staff have a positive impact on maintaining mental health (11).

Therefore, we explored the mental health outcomes of healthcare workers caring for patients with COVID-19 in Fangcang shelter hospitals in Wuhan. To improve the validity of our research, we made a questionnaire to investigate 240 patients from the Wuhan hospital. We also provided additional evidence with respect to the mental wellbeing of healthcare workers. In addition, we used Patient Health Questionnaire-9 items Scale (PHQ-9) to evaluate the severity of the participants' depressive symptoms and Generalized Anxiety Disorder-7 items Scale (GAD-7) to assess the severity of the participants' anxiety symptoms.

## Method

### Participants and Sampling

This study was cross-sectional designed and conducted between February and March 2020, ~1.5 months after the novel coronavirus disease 2019 (COVID-19) outbreak. A total of 240 participants recruited were medical workers from Fangcang shelter hospitals located in Wuhan city in China. The participants were allowed to finish the survey after the closure of Fangcang shelter hospitals. A total of 240 medical staff members completed the questionnaires voluntarily. The average working time of the participants in the Fangcang shelter hospital was 21.73 ± 10.69 days, 189 people (78.8%) were from Wuhan Living Room Fangcang Shelter Hospital, and 51 (21.2%) were from other Fangcang shelter hospitals. The average age of the participants was 35.27 ± 7.31 years old, including 159 women (66.3%), 81 men (33.8%), 82 doctors (34.2%), 143 nurses (59.6%), and 15 other staff (6.3%). Of 189 people, 58 individuals (24.2%) have previously participated in medical emergency rescue missions. In terms of mental health, 83 people (34.6%) had depression symptoms, and 93 people (38.8%) had anxiety symptoms. Univariate analysis showed that people who had not participated in medical emergency rescue missions before were more prone to depression symptoms (χ^2^ = 8.035, *P* < 0.05). The healthcare workers with insufficient training before entering Fangcang shelter hospitals had a greater proportion of depression and anxiety symptoms (*P* < 0.05).

In accordance with the Declaration of Helsinki, the ethics panel of the Medical Association of Shanghai East Hospital approved this study (No. 2020007). All the participants provided informed consent for research details and anonymized responses to be published. They were clearly informed that they had the right to refuse or withdraw from the research at any time. In the light of standardized instructions, we introduced the research purpose, questionnaire content, study process, and principle of confidentiality to the potential participants. The survey was anonymous, and because of the requirements of epidemic prevention, informed consent was provided electronically by all the survey participants after participating in the study.

### Assessment Procedure

The survey participants used a mobile phone or a tablet to scan a WeChat QR code to access the mobile questionnaire survey system. All the participants filled in the questionnaires and scales online, which consisted of a general information questionnaire, a questionnaire on Fangcang shelter hospital work, the 9-item Patient Health Questionnaire Scale (PHQ-9), and the 7-item Generalized Anxiety Disorder Scale (GAD-7).

### Questionnaire

This questionnaire included age, gender, occupational role (physician, nurse, or other), marital status, educational level (< undergraduate, bachelor or >graduate), types of personality traits, and past history of mental illness. This questionnaire was self-reported by the participants, including items related to the Fangcang shelter hospitals' scope of work, such as training and mental preparation prior to entering hospital, stress factors before work, past experience in emergency rescue, and coping methods.

### Depression and Anxiety Assessment

With Chinese versions of validated measurement tools, symptoms of depression and anxiety among all the participants are investigated. As such, PHQ-9 and GAD-7 were used to assess the degree of severity of depression and anxiety symptoms, respectively. The outcome scores of measurement tools were interpreted as follows: the two scales' categories were both based on established values in the literature, and items were rated on a 4-point Likert-type scale, ranging from 0 (not at all) to 3 (nearly every day). Total scores of PHQ-9 range from 0 to 27, with high scores meaning more depression symptoms. Based on existing original validation studies, the outcome of total scores was interpreted as four symptom levels suggesting no depression (0–4), mild (5–9), moderate (10–14), and severe (15–27), separately. Total scores of GAD-7 range from 0 to 21, with high scores meaning more anxiety symptoms of four levels of classification, which suggest no anxiety (0–4), mild (5–9), moderate (10–14), and severe (15–21), separately.

### Investigation on Psychological Adjustment Methods

Nine self-help psychological adjustment methods were investigated for the participants, including communication with family members and friends, communication with colleagues in the Fangcang shelter hospital, physical exercise, typical recreational activities (such as reading, watching videos, listening to music, and playing games), reading books that guide psychological adjustment (either paper or Ebook), listening to an audio guide to psychological adjustment, self-learning self-relaxation techniques (such as breathing adjustments and progressive muscle relaxation exercises) through the Internet, calling the psychological assistance hotline, and seeking help from a psychological specialist (online or offline).

### Statistical Analysis

SPSS 22.0 (IBM, Chicago) was used for statistical analysis. The significance level was set at α = 0.05, and all tests were 2-tailed. Descriptive statistics were computed for categorical variables, and a Chi-squared test compared their respective rate variation. The continuous data of this questionnaire were not normally distributed and therefore presented as medians with interquartile ranges (IQRs). The non-parametric Mann-Whitney U test and the Kruskal-Wallis test were conducted to compare the severity of symptoms between two or more groups. To explore potential risk factors for symptoms of depression and anxiety in the participants, a logistic regression analysis was performed, and the risk factors are presented as odds ratios (ORs) and 95% confidence intervals (CIs), with adjustment for confounders, namely, age, gender, marital status, educational level, emergency rescue experience, and degree of training.

## Result

In the study, 240 participants volunteered to complete the survey, 82 were physicians, 143 were nurses, and 15 were miscellaneous staff (such as laboratory personnel). Majority of the participants were women (159) aged 30–40 years (134), were married (180), had an educational level of undergraduate (153), and worked for 2–4 weeks in the Fangcang shelter hospital (Table 1).

**Table 1:** Demographic characteristics of participants.

**Variable N**	**Total (%)**	**Physicians (%)**	**Nurses (%)**	**Other staffs (%)**
Overall	240 (100)	82 (34.2)	143 (59.6)	15 (6.3)
**Gender**				
Men	81 (33.8)	57 (69.5)	13 (9.1)	11 (73.3)
Women	159 (66.3)	25 (30.5)	130 (90.9)	4 (26.7)
**Marriage status**				
Unmarried	60 (25.0)	9 (11.0)	48 (33.6)	3 (20)
married	180 (75)	73 (89.0)	95 (66.4)	12 (80)
**Education level**				
< Undergraduate	40 (16.7)	2 (2.4)	36 (25.2)	2 (13.3)
=bachelor	153 (63.8)	39 (47.6)	104 (72.7)	10 (66.7)
>Graduate	47 (32.9)	41 (50.0)	3 (2.1)	3 (20.0)
**Age** (**years)**				
<30	55 (22.9)	5 (6.1)	46 (32.2)	4 (26.7)
30–40	134 (55.8)	43 (52.4)	85 (59.4)	6 (40.0)
>40	51 (21.3)	34 (41.5)	12 (8.4)	5 (33.3)
**Length of work (years)**				
≤ 10	115 (47.9)	31 (37.8)	79 (55.2)	5 (33.3)
11–20	80 (33.3)	22 (26.8)	49 (34.3)	9 (60.0)
>20	45 (18.8)	29 (35.4)	15 (10.5)	1 (6.7)
**Weeks in the Fangcang shelter hospital**				
≤ 2	73 (30.4)	26 (31.7)	37 (25.9)	10 (66.7)
2–4	114 (47.5)	29 (35.4)	83 (58.0)	2 (13.3)
>4	53 (22.1)	27 (32.9)	23 (16.1)	3 (20)
**Emergency rescue experience**				
Yes	58 (24.2)	26 (31.7)	25 (17.5)	7 (46.7)
No	182 (75.8)	56 (68.3)	118 (82.5)	8 (53.3)

### Physicians vs. Nurses and Subgroup Analysis

Differences between the physicians and the nurses in terms of demographic characteristics, namely, gender, age, education level, marriage, and length of work, were statistically significant (*P* < 0.05). Most of the doctors are male, older, married, and have higher education levels and longer working years.

According to this study, the physicians and nurses working in Fangcang shelter hospitals vary in terms of personal background. The physicians (31.7 vs. 17.5%) had more experience in rescue missions than the nurses (*P* < 0.05). However, the nurses trumped the physicians in terms of support from family members (66.4 vs. 48.8%) to work in Fangcang shelter hospitals. Among the physicians, highly educated physicians were more likely to worry about being infected (85.4 vs. 61%); young physicians are more worried about being incapable of working in the Fangcang shelter hospital and having a heavy workload (*P* < 0.05).

Among physicians, those who had an above bachelor's degree were more likely to be stressed for the probability to be infected. Speaking to family was the method of choice of more educated doctors for psychological self-adjustment compared with the counterpart with less academic education. Physicians with above bachelor's degrees showed higher rates of depression and anxiety symptoms (Table 2). There were no similar results among the nurses.

**Table 2:** Subgroup physicians (*n* = 82).

**Category** ***N*** **(%)**	**Undergraduate and below *n* = 41**	**Above bachelor's degree *n* = 41**	***p***
Worries being infected before work	25 (61.0)	35 (85.4)	0.013
Talk to family for psychological adjustment	26 (63.4)	36 (87.8)	0.010
Depression	10 (24.4)	21 (51.2)	0.012
Anxiety	9 (22.0)	18 (43.9)	0.034

*Fisher exact method.

### With Past Emergency Rescue Experience vs. Without Past Emergency Rescue Experience

Compared with those who had emergency rescue experience, participants with no emergency rescue experience were more nervous about overload (before they went to Fangcang shelter hospital [38 (20.9%) vs. 5 (8.6%), *p* = 0.03]. Those with emergency rescue experience were more worried about being infected (Z= −3.557, *p* < 0.05 and the health of their families (Z = −3.056, *p* < 0.05) during their Fangcang shelter hospital tenure. The difference between PHQ-9 and GAD-7 scores was not statistically significant.

### Risk Factors of Depression and Anxiety

The logistic regression analysis identified the following two groups to be at a higher risk of developing depression symptoms: no emergency rescue experience and inadequate training. In addition, the logistic regression analysis showed that the individuals with inadequate training were at higher risk of experiencing anxiety symptoms (Table 3).

**Table 3:** Risk factors for depressive and anxiety symptom.

**Variable**	**OR**	**95%CI**	***p***
**PHQ-9**			
No emergency rescue experience	2.610	1.398–4.872	0.003
Inadequate training	2.804	1.293–6.080	0.009
**GAD-7**			
Inadequate training	2.692	1.300–5.575	0.008

*Binary logistic regression.

### Psychological Adjustment Methods and Self-Evaluation Effectiveness

The top three frequently used methods of psychological adjustment for the staff in the Fangcang shelter hospitals are usual entertainment (such as reading, watching videos, listening to music, and playing games) (93.8%), communicating with colleagues in the Fangcang shelter hospital (92.5%), and communicating with family members and friends (78.3%), and the three most effective methods of psychological adjustment were calling the psychological assistance hotline (81.8%), seeking help from a psychological specialist (64.3%), communicating with colleagues in the Fangcang shelter hospital 120 (54.1%) (Table 4).

**Table 4:** Psychological adjustment method and Self-evaluation effectiveness.

**Psychological adjustment method**	**Number of options *n* (%)**	**Self-evaluation effectiveness** ***n*** **(%)**			
		**Very helpful**	**Helpful**	**Help a bit**	**Helpless**
1. Communicating with family members and friends	188 (78.3)	99 (52.7)	71 (37.8)	15 (8.0)	3 (1.6)
2. Communicate with colleagues in the Fangcang shelter hospital	222(92.5)	120 (54.1)	82 (36.9)	14 (6.3)	6 (2.7)
3. Physical exercise	168 (70.0)	78 (46.4)	71 (42.3)	12 (7.1)	7 (4.2)
4. Usual entertainment	225 (93.8)	105 (46.7)	103 (45.8)	12 (5.3)	5 (2.2)
5. Read books that guide psychological adjustment	132 (55.0)	63 (47.7)	58 (43.9)	8 (6.1)	3 (2.3)
6. Listen to an audio guide to psychological adjustment	92 (38.3)	46 (50.0)	36 (39.1)	9 (9.8)	1 (1.1)
7. Self-learning self -relaxation techniques	110 (45.8)	53 (48.2)	45 (40.9)	9 (8.2)	3 (2.7)
8. Call the psychological assistance hotline	11 (4.6)	9 (81.8)	0	1 (9.1)	1 (9.1)
9. Seek help from a psychological specialist	14 (5.8)	9 (64.3)	4 (28.6)	0	1 (7.1)

## Discussion

This study found that there was no variance in anxiety and depression levels between the physicians and the nurses. Previous research shows that women, nurses, frontline healthcare workers, and those working in Wuhan reported significantly higher severity of mental health symptoms than all other healthcare workers. The frontline healthcare workers who engaged in the daily work of diagnosis, treatment, and nursing of patients with COVID-19 had a higher risk of experiencing symptoms of insomnia, anxiety, depression, and distress (4). Other research shows that being at risk of contact with patients with COVID-19, living in rural areas, and being female were the most common risk factors for people developing insomnia, anxiety, depression, and obsessive-compulsive symptoms (5). The possible rationale might be fear of infection, prolonged working hours, lack of protective medical apparatus, infected patient load, less effective COVID-19 medication, loss and death of medical colleagues after exposure to COVID-19, social distancing, or isolation from family and friends, and the deteriorating situation of patients may have a negative impact on the mental health of medical workers (12).

Compared with those of the physicians, the nurses' families were more supportive of their work in the Fangcang shelter hospital [40 (48.8%) vs. 95 (66.4%), *p* = 0.03]. There were no differences in scores measuring symptoms of depression and anxiety between the two groups. Those who had experiences of previous rescue missions were more worried about getting infected and the health status of their family members. By regression analysis, it was found that depression and anxiety symptoms were related to whether they had participated in rescue training and sufficient pre-go-live training.

Research shows receiving negative information about COVID-19 and participating in frontline work against COVID-19 appear to be vital risk factors for mental health problems (13–15). In terms of psychological adjustment, this study demonstrated that doctors with higher education levels had more contacts with their family members (87.8 vs. 63.4%); older doctors were more inclined to adjust their psychological pressure by reading (Z = −1.99, *P* < 0.05). Among the nurses, even the older nurses were more worried about being infected (Z = −2.076, *P* < 0.05), but they were more inclined to adjust their psychological stress through physical exercise (Z = −3.001, *P* < 0.05). Based on these investigation results, healthcare workers have sufficient resources at their disposal to overcome the stress related to their daily work in the Fangcang shelter hospital (16).

This result shows that within a centralized isolation treatment environment of the Fangcang shelter hospital, stress is widely existent, and depression and anxiety symptoms are common. For the medical staff in Fangcang shelter hospitals, training before on-boarding, appropriate recreational activities, and strengthening interpersonal communication and support can help reduce the stress, anxiety, and depression levels.

## Conclusion

In conclusion, we found that, for the mental health of medical personnel fighting COVID-19, it is very important for them to have medical rescue experience and undergo pre-go-live training. With the psychological adjustment methods of improving interpersonal contact and professional help and peer support, medical workers can regain their psychological strength against stress from COVID-19.

However, this research is limited in time frame and because of the cross-sectional design. The psychological assessment was based on a voluntary online survey and self-report tools and had a small sample size. Future longitudinal studies are required for follow-up of the mental wellbeing of medical workers. Qualitative research interviews are also encouraged in future studies to generate a more comprehensive understanding of follow-up mental wellbeing adjustment (e.g., Balint group).

## Data Availability Statement

The original contributions presented in the study are included in the article/supplementary material, further inquiries can be directed to the corresponding author/s.

## Ethics Statement

The ethics panel of the Medical Association of Shanghai East Hospital approved this study (No.2020007). All the participants provided informed consent for research details and anonymized responses to be published. They were clearly informed that they had the right to refuse or withdraw from the research at anytime. In the light of standardized instructions, we introduced the research purpose, questionnaire content, study process, and principle of confidentiality to the potential participants. The survey was anonymous, and because of the requirements of epidemic prevention, informed consent was provided electronically by all the survey participants after participating in the study.

## Author Contributions

QF and CK: conception and design. QF, HZ, and LW: data analysis and interpretation. All authors: administrative support, provision of study materials or patients, collection and assembly of data, manuscript writing, and final approval of manuscript.

## Funding

This study was supported by the Special Fund for Fighting the COVID-19 Outbreak sponsored by Tongji University School of Art and Media and Institute of Disaster Medicine, East Hospital, Tongji University (20TJBXKY06).

## Conflict of Interest

The authors declare that the research was conducted in the absence of any commercial or financial relationships that could be construed as a potential conflict of interest.

## Publisher's Note

All claims expressed in this article are solely those of the authors and do not necessarily represent those of their affiliated organizations, or those of the publisher, the editors and the reviewers. Any product that may be evaluated in this article, or claim that may be made by its manufacturer, is not guaranteed or endorsed by the publisher.

## References

[1] China NHCo. New Coronavirus Pneumonia Diagnosis Treatment Plan (Trial version 7). (2020). Available online at: http://wwwnhcgovcn/yzygj/s7653p/202003/46c9294a7dfe4cef80dc7f5912eb1989/files/ce3e6945832a438eaae415350a8ce964pdf (accessed June 17, 2021).

[2] Chen S Yang J Yang W Wang C Bärnighausen T. COVID-19 control in China during mass population movements at New Year. Lancet. (2020) 395:764–66. 10.1016/S0140-6736(20)30421-9 32105609 PMC7159085

[3] The State Council of the People's Republic of China. The Joint Prevention and Control Mechanism of the State Council Launch Announcement on Further Shouldering Responsibilities and Implementing Prevention and Control Strategies (in Chinese). (2020). Available online at: http://www.gov.cn/guowuyuan/2020-02/07/ content_5475951.htm (accessed February 24, 2020).

[4] Lai J Ma S Wang Y Cai Z Hu J Wei N. Factors associated with mental health outcomes among health care workers exposed to coronavirus disease 2019. JAMA Network Open. (2020) 3:e203976. 10.1001/jamanetworkopen.2020.3976 32202646 PMC7090843

[5] Zhang WR Wang K Yin L Zhao WF Xue Q Peng M. Mental health and psychosocial problems of medical health workers during the COVID-19 epidemic in China. Psychother Psychosom. (2020) 89:242–50. 10.1159/000507639 32272480 PMC7206349

[6] WHO. WHO Coronavirus Disease (COVID-19) Situation Dashboard. (2021). Available online at: https://covid19whoint/ (accessed June 17, 2021).

[7] WHO. Coronavirus Disease (COVID-19). (2021). Available online at: https://wwwwhoint/emergencies/diseases/novel-coronavirus-2019/question-and-answers-hub/q-a-detail/q-a-coronaviruses (accessed June 17, 2021).

[8] Liu Q Luo D Haase JE Guo Q Wang XQ Liu S. The experiences of health-care providers during the COVID-19 crisis in China: a qualitative study. Lancet Global Health. (2020) 8:E790–8. 10.1016/S2214-109X(20)30204-7 32573443 PMC7190296

[9] Chinanews. Beijing: Two Departments Issue the Novel Coronavirus Infection Pneumonia Diagnosis and Treatment Plan. (trial version 7). Available online at: http://www.chinanews.com/gn/2020/03-04/9113100.shtml. Chinese (accessed March 4, 2020).

[10] SINAnews. Beijing: In January, Hubei Had More Than 3,000 Medical Infections, and the Wuhan Health and Medical Committee Reported “None” for Half a Month. Available online at: https://news.sina.com.cn/o/2020-03-06/dociimxyqvz8395569.shtml. Chinese (accessed March 6, 2020).

[11] Wang Y Zhao X Feng Q Liu L Yao Y Shi J. Psychological assistance during the coronavirus disease 2019 outbreak in China. J Health Psychol. (2020) 25:135910532091917. 10.1177/1359105320919177 32301628

[12] Mental Health and Coping During COVID-19. Centers for Disease Control and Prevention (2020). Available online at: https://www.cdc.gov/coronavirus/2019-ncov/about/coping.html (accessed May 03, 2020).

[13] Que J Le Shi JD Liu J Zhang L Wu S Gong Y. Psychological impact of the COVID-19 pandemic on healthcare workers: a cross-sectional study in China. Gen Psychiatry. (2020) 33:e100259. 10.1136/gpsych-2020-100259 32596640 PMC7299004

[14] Fang D Pan S Li Z Yuan T Jiang B Gan D. Large-scale public venues as medical emergency sites in disasters: lessons from COVID-19 and the use of Fangcang shelter hospitals in Wuhan, China. BMJ Global Health. (2020) 5:e002815. 10.1136/bmjgh-2020-002815 32546589 PMC7298718

[15] Dai LL Wang X Jiang TC Li PF Wang Y Wu SJ. Anxiety and depressive symptoms among COVID-19 patients in Jianghan Fangcang Shelter Hospital in Wuhan, China. PLoS ONE. (2020) 15:e0238416. 10.1371/journal.pone.0238416 32857826 PMC7454940

[16] National Health Commission of the People's Republic of China. Press conference of the joint prevention and control mechanism of the State Council on Feb 29, 2020. National Health Commission of the People's Republic of China (2020). Available online at: http://www.nhc.gov.cn/xwzb/webcontroller.do?titleSeq=11248&gecstype=1 (accessed March 1, 2020).

